# High infection rate of zoonotic *Eucoleus aerophilus* infection in foxes from Serbia

**DOI:** 10.1051/parasite/2012003

**Published:** 2013-01-14

**Authors:** Vesna Lalošević, Dušan Lalošević, Ivan Čapo, Verica Simin, Annamaria Galfi, Donato Traversa

**Affiliations:** 1 Faculty of Agriculture, University of Novi Sad Trg Dositeja Obradovica 8 21000 Novi Sad Serbia; 2 Faculty of Medicine, University of Novi Sad Hajduk Veljkova 3 21000 Novi Sad Serbia; 3 Pasteur Institute Hajduk Veljkova 1 21000 Novi Sad Serbia; 4 Department of Comparative Biomedical Sciences, University of Teramo Teramo Italy

**Keywords:** *Eucoleus aerophilus*, *Eucoleus boehmi*, red fox, Serbia

## Abstract

The respiratory capillariid nematode *Eucoleus aerophilus* (Creplin, 1839) infects wild and domestic carnivores and, occasionally, humans. Thus far, a dozen of human infections have been published in the literature but it cannot be ruled out that lung capillariosis is underdiagnosed in human medicine. Also, the apparent spreading of *E. aerophilus* in different geographic areas spurs new studies on the epidemiology of this nematode. After the recognition of the first human case of *E. aerophilus* infection in Serbia, there is a significant merit in enhancing knowledge on the distribution of the nematode. In the present work the infection rate of pulmonary capillariosis was investigated in 70 red foxes (*Vulpes vulpes*) from the northern part of Serbia by autopsy. The estimated infection rate with *Eucoleus aerophilus* was 84%. In contrast, by copromicroscopic examination only 38% of foxes were positive. In addition, 10 foxes were investigated for the closely related species in nasal cavity, *Eucoleus boehmi*, and nine were positive. Our study demonstrates one of the highest infection rates of pulmonary capillariosis in foxes over the world.

## Abstract (fr)

Le nématode Capillariidae de l’appareil respiratoire *Eucoleus aerophilus* (Creplin, 1839) infecte les carnivores sauvages et domestiques et parfois les humains. À ce jour une douzaine d’infections humaines ont été publiées dans la littérature, mais il ne peut pas être exclu que la capillariose du poumon soit sous-diagnostiquée en médecine humaine. En outre, l’apparente propagation de *E. aerophilus* dans différentes zones géographiques stimule de nouvelles études sur l’épidémiologie de ce nématode. Après la reconnaissance du premier cas humain d’infection à *E. aerophilus* en Serbie, il est important d’améliorer les connaissances sur la distribution de ce nématode. Dans le présent travail le taux d’infection de capillariose pulmonaire a été étudié par autopsie chez 70 renards roux (*Vulpes vulpes*) du nord de la Serbie. Le taux d’infection à *E. aerophilus* estimé était de 84 %. En revanche, seulement 38 % des renards étaient positifs par examen copromicroscopique. En outre, dix renards ont été étudiés pour l’espèce étroitement apparentée des fosses nasales *Eucoleus boehmi*, et neuf ont été positifs. Notre étude démontre l’un des taux d’infection le plus élevés au monde de capillariose pulmonaire chez les renards.

## Introduction

Nematodes of the family Capillariidae Neveu-Lemaire, 1936 are a very large group with not yet completely accepted taxonomy [16, 32]. Among species ranked in this family, *Eucoleus aerophilus* (Creplin, 1839) Dujardin, 1845 (syn. *Capillaria aerophila* or *Thominx aerophilus*) affects trachea and main bronchi of canids, felids and some omnivorous animals [19]. Although several morphological descriptions have been provided for different biological stages of *E. aerophilus* [3, 5, 18, 23, 32], several other aspects of this nematode are still poorly known. Indeed, this is the case of geographic distribution, clinical significance and actual zoonotic potential [31]. Also, knowledge on the biological cycle of this nematode is scanty and, for instance, the route of transmission to vertebrate hosts remains to be understood. In general it is thought that animals become infected by ingesting environmental larvated eggs [31] but it has been also hypothesized, but never demonstrated, that larval *E. aerophilus* might require the passage through earthworms to become infective for cats and foxes (Borovkova, 1947 cited in [18]).

In the past few years some evidences have suggested that *E. aerophilus* is spreading in several geographic areas and that there is an increase of cross-infections between wild and domestic animals [7–9, 30, 31]. Indeed, it has been preliminarily shown that some *E. aerophilus* populations are shared between domestic pets and wildlife, e.g. red fox (*Vulpes vulpes*) and beech-marten (*Martes foina* (Erxleben) [9]).

Such changes are a likely effect of the increase of fox populations in peri-urban and urban areas and of movements of wild and companion animals around regions. In fact, an increase of the red fox population has been documented in Europe after the success of the oral vaccination against rabies [4, 33]. The role of the red fox as reservoir for parasites may be now of relevance especially in suburban areas, e.g. for multilocular echinococcosis and other parasitic diseases of veterinary (e.g. angiostrongylosis) and human (e.g. trichinellosis) concern [11, 15, 21, 22, 31].

With regard to Serbia, *E. aerophilus* is circulating in cats and foxes [12, 13] and, interestingly, the last case of human capillariosis documented in the literature is from the same country. More specifically, an infected woman showed respiratory symptoms miming bronchial carcinoma and blood eosinophilia [12]. Considering all the aforementioned background, especially the potential risk for human health which causes new interest in capillariid nematodes, the aim of this work was to investigate the infection rate of *E. aerophilus* in red foxes of Serbia.

## Materials and Methods

### Study area

In winter season from 2008 to the end of 2011, foxes (*n* = 118) hunted in the framework of a rabies control campaign were collected from the whole territory (21,506 square km) of the Vojvodina province of Serbia (45°15 N, 19°50′ E). This province is crossed by the Danube river, two tributaries (Tisza and Sava) and many canals of the Danube-Tisza-Danube system (Pannonian Basin) on the left side and the Fruska Gora mountain on the right. The climate is continental.

### Sampling

All foxes were adults with a body weight ranging from 4.0 to 8.1 kg. Carcasses were opened and the tracheas of 70 foxes, from the larynx to the main bronchi bifurcation, were collected and preserved in 30% ethanol. Tracheas were then opened on the anterior side by scissors and parasites retrieved were collected under a stereomicroscope and wet-mounted in glycerine-ethanol or lactophenol. The nasal cavity of 10 foxes was opened through the palate, and the nasal mucosa was scrapped and conserved in 30% ethanol. Faecal samples were collected during the necropsy of the 118 foxes and tested by the glycerine flotation method, respectively. Parasites collected were counted, photographed by a Leica microscope and measured by the “ImageJ” free program (http://imagej.nih.gov/ij/). Adult stages of nematodes recovered from trachea and bronchi of the foxes were identified at the species level by morphological keys [17, 23]. Five adult female and three male *E. aerophilus* voucher specimens were deposited in the Zoological collection of the Department of Biology and Ecology, University of Novi Sad, Serbia (Registration No. NEM-0037).

## Results

**Figure 1.**
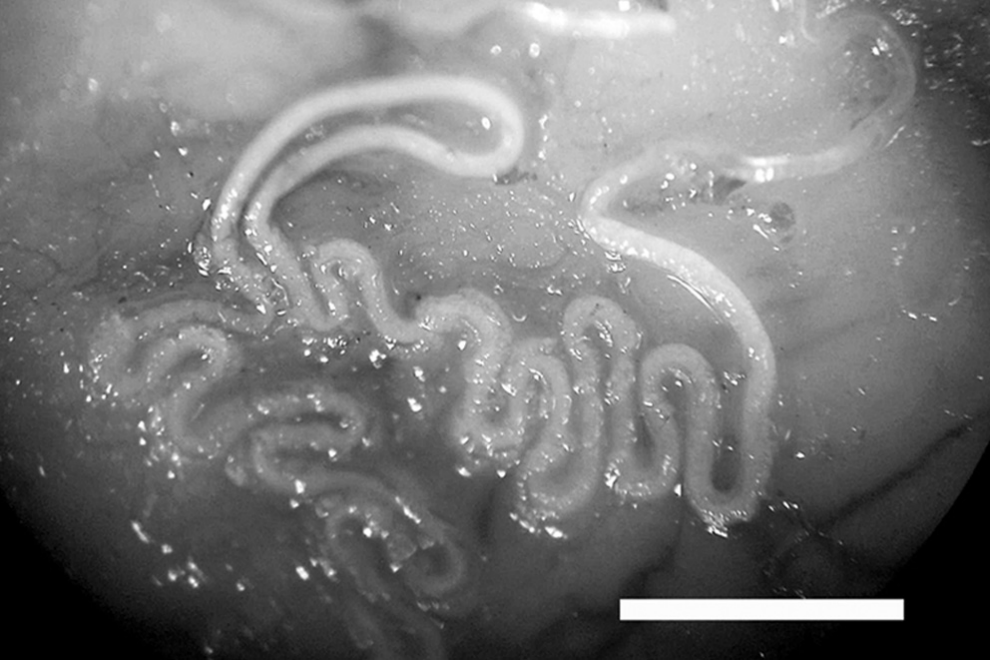
Adult female *Eucoleus aerophilus* on the mucosa of the opened trachea of a fox dissected after formalin fixation. More than half of the worm body is inside the tracheal mucosa with a zig-zag shape and the other part is free in the lumen. Bar, 1 mm.

**Figure 2.**
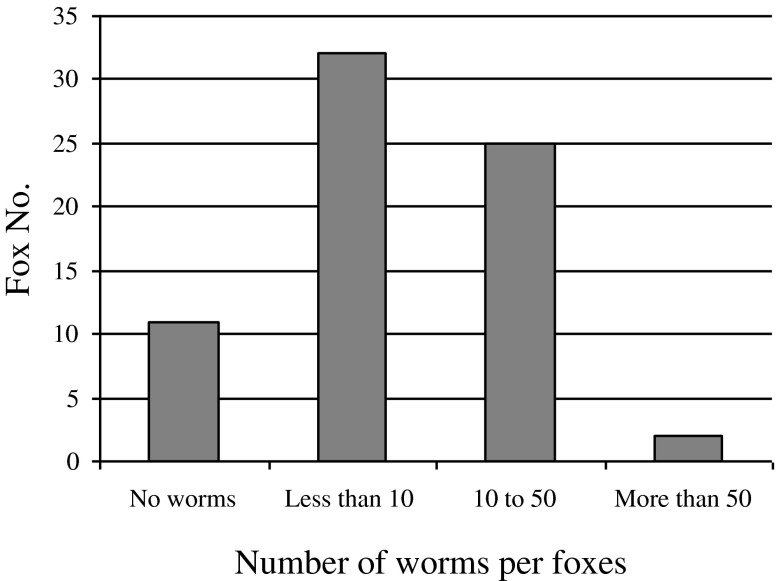
Number of *Eucoleus aerophilus* adults per foxes.

Adults of *E. aerophilus* (i.e. total of 817) were detected in 59 foxes (84%), with an average worm burden of 14 (range 1–71) (Figures 1 and 2). Out of 70 examined foxes, 32% harboured more than 14 adult worms of *E. aerophilus* with no relevant difference in parasitic burden between foxes collected from different mountainous regions or the Pannonian basin. The sex was assessed for 390 out of 817 examined worms because some *E. aerophilus* specimens were damaged during the collection. The sex ratio between male and female worms was 1:2.56.

**Figure 3.**
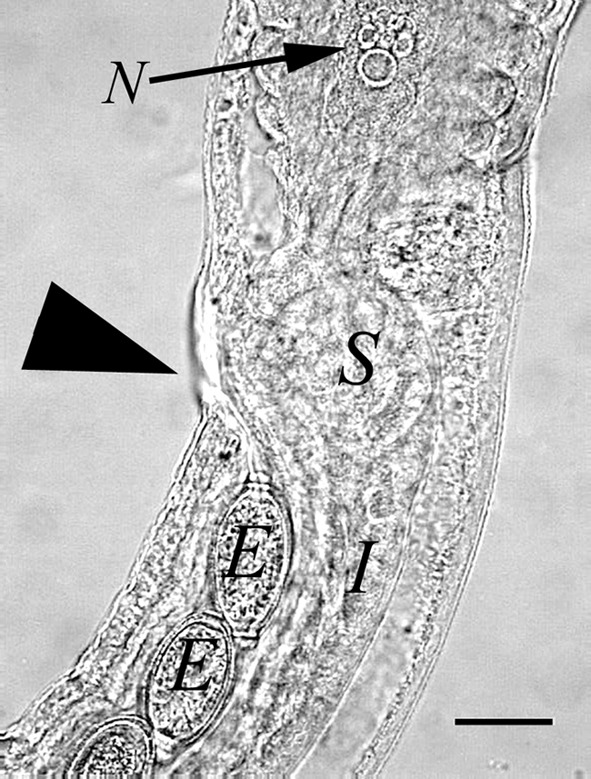
Female *Eucoleus aerophilus* showing the end part of the last stichocyte with the cell nucleus (N), two superimposed secretory cells (S), intestine (I), vagina with eggs (E) and vulva (arrowhead). Bar, 50 μm.

**Figure 4.**
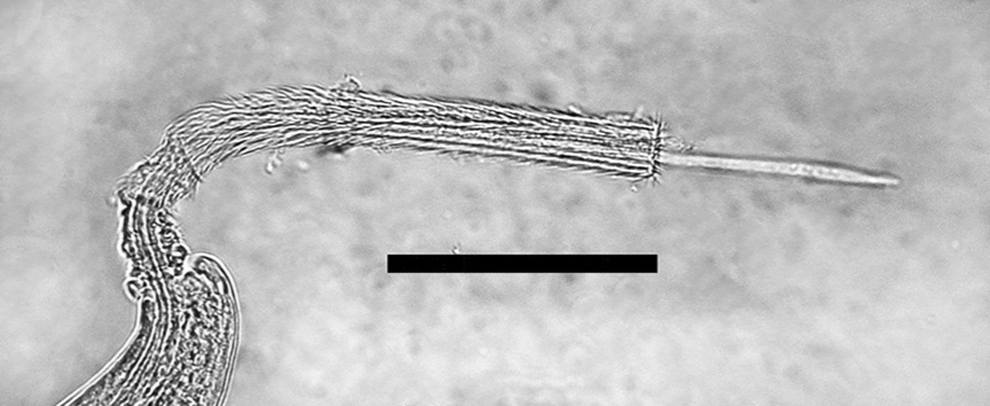
Male *Eucoleus aerophilus*, extruded spinose spicule sheath and partially extruded spicule at the caudal part. Bar, 100 μm.

Morphometric measures were obtained from 82 males and 75 females. Measurements are in micrometer (μm) and indicated here as mean and range between parentheses. Body length, male 19,020 (10,410–25,110), female 27,980 (16,010–41,840); mean diameter measured after end of oesophagus, male 86 (69–110), female 147 (107–185); oesophagus length, male 6,590 (4,390–7,980), female 6,003 (4,540–7,330); number of stichocytes, calculated in 10 males and 11 females, male 44 (37–46), female 44.5 (35–50). The lateral bacillary bands were well recognized but the ventral bacillary bands were observed rarely. The uterus containing numerous eggs continued in a muscular vagina with a vulvas opening (Figure 3). Eggs were unembryonated, thick-shelled with a reticulated surface and with polar plug-like opercula, giving them a lemon shape. Eggs not symmetrical, opercular axes slightly angulated. Measurements of eggs in the final part of the uterus of 75 females: 73 × 35 (61–95 × 30–47). The caudal end of males showed a small pseudobursa with two bulges next to the cloaca opening. Males showed a single very thin spicule and a spicule sheath with many spines (Figure 4).

For 10 foxes the nasal cavity mucosa was scrapped and examined under a stereomicroscope. Adult stages belonging to the closely related species *Eucoleus boehmi* (Supperer, 1953) were found in nine animals. The average worm burden was seven (range 1–20).

The results of the copromicroscopic examinations on faecal samples collected at the necropsy are reported in Table 1.

**Table 1.** Results for the copromicroscopic examinations of the foxes (*n* = 118) examined in the present study. *N*: number of foxes scored positive for endoparasites.

Parasite species	*N*	Infection rate (%)
*Toxascaris leonina*	44	37
*Toxocara canis*	19	16
*Trichuris vulpis*	13	11
*Ancylostoma* spp.	29	25
Capillariidae gen*.* spp.	45	38
*Isospora* spp.	1	0.84
Parasite free	23	20

## Discussion

The infection rate of *E. aerophilus* infection (84%) herein detected in foxes from the Pannonian and the Fruska Gora Mountain regions (Vojvodina province) is indeed high. Interestingly, along with the results (88%) recently obtained from foxes of Norway [6], this study demonstrated the highest infection rate of pulmonary capillariosis in wild canids in Europe.

In the Zagreb region (Croatia) which shows a similar landscape to the central part of Serbia, about 300 km on the West, the prevalence of *E. aerophilus* in foxes was only 4.7% with a highest worm burden of five worms [20]. In Italy, the infection rate of *E. aerophilus* detected in the trachea of foxes was 7.0% in the Tuscany region [14]. In the Netherlands and Hungary *E. aerophilus* was found in 46.8% and 66% of dissected foxes [1, 27]. An infection rate by “*Capillaria* spp.” of 22.4% was found in foxes from Slovakia by copromicroscopic investigation [15].

Albeit the high prevalence of *E. aerophilus* in foxes from Serbia could be due to the high sensitivity of the diagnostic methods used, most of the aforementioned studies have relied on trachea dissection as well. A great discrepancy between copromicroscopic and necroscopical findings of capillariids in the present study demonstrates that copromicroscopy may fail in terms of diagnostic sensitivity for this infection in foxes. Therefore, factors other than the diagnostic approach should be implicated in influencing such a high infection rate by lung capillariosis in foxes from Serbia.

The high humidity of the habitat of Vojvodina, the South part of Pannonian Basin, with many canals between the Danube and Tisza rivers, and many small tributaries of the Sava river in the Fruska Gora Mountain could favour parasite’s dissemination. In this habitat, the fox density is of over 0.2 per square km and the high humidity can favour earthworms’ dispersion. Also, competitive pressure and other diseases (e.g. sarcoptic mange) can enhance the susceptibility of foxes to nematodes [24] and the increase of reports of capillariosis in companion animals suggests that *E. aerophilus* is spreading in several areas. Epidemiological data (e.g. range of hosts and geographic distribution) of *E. aerophilus* in Europe are poorly known, thus at the moment it is difficult to assess to what degree this parasite may be spreading or what influence global warming or other factors may have on the current distribution [31]. On the other hand, it is known that wild foxes act as reservoirs and amplifiers of several canid nematodes, thus they can re-enforce environmental contamination and risk of infection for domestic dogs and humans as well [29].

There is still not an agreement of the taxonomy of capillariid nematodes. Key morphological characters (i.e. eggs, oesophageal structure and genital organs of adult stages) allow the identification of *E. aerophilus* but, sometimes its identification at the species level and the differentiation from *E. boehmi* may be difficult.

While Christenson [5] did not detect spicule and spicule sheath in male *E. aerophilus*, more recent line drawings of this very characteristic organ were published by Butterworth and Beverley-Burton [3], Moravec [17] and Romashov [23]. The present work confirms the presence of these morphological features, which is of key diagnostic relevance for the identification at the species level of male *E. aerophilus*.

The stichocyte number, higher in *E. aerophilus* than in *E. boehmi*, can help to distinguish these two species between them, even though in some cases, the number of stichocytes may overlap. In fact in the present study, one *E. boehmi* female showed 35 stichocytes as an *E. aerophilus* female. According to Moravec [17], *E. boehmi* and *E. aerophilus* females have 30–32 and 42–46 stichocytes, respectively, whereas, according to Romashov [23], the number of stichocytes in the female is 32–36 for *E. boehmi* and 35–49 for *E. aerophilus*. Greater variations of the stichocyte number were reported in *E. aerophilus* males, i.e. 42–55 [23], or 43–50 [17]. The present study revealed that the number of stichocytes may be useful in the identification of *Eucoleus* spp. but it is not absolute criteria. The localization of adult stages could also greatly help in distinguishing between *E. aerophilus* (in trachea and bronchi) and *E. boehmi* (in nasal cavities).

Another issue warranting further investigations is the size of the eggs. While some textbooks report a length of up to 83 μm for *E. aerophilus* eggs, e.g. Soulsby [26], Bowman *et al.* [2], Taylor *et al.* [28], recent studies have demonstrated that the length of *E. aerophilus* eggs from dogs and cats is unwaveringly less than 70 μm [7, 8, 32]. Interestingly, some ova collected from the uterus of adult *E. aerophilus* in the present study were found to be longer and this could be from different reasons. It could be that eggs undergo further development and modifications when they are passed with the faeces, or the existence of different morphotypes within the same species *E. aerophilus* and with different host affiliations could be hypothesized. Recent molecular studies have shown that different haplotypes of *E. aerophilus* may be either shared between domestic and wild carnivores or are affiliated to specific hosts [9]. Further studies are necessary for evaluating the distribution of *E. aerophilus* in wildlife and pets cohabiting the same geographic areas in order to elucidate the phylogeography of different parasite populations and for verifying the possible existence of different morphotypes [8, 9].

Under a clinical standpoint, the pathogenic role of *E. aerophilus* in wildlife is not well recognized because it is ranging from low to high and *E. aerophilus* has been also considered as an agent of massive mortality in farmed silver foxes [25]. In the present work, a very low inflammatory response was found around the worms (data not shown) which can be the result of a low pathogenicity or an immunosuppression in the mucosa caused by the parasite in these animals. A past work carried out in six experimentally infected foxes showed that animals may develop bronchopneumonia with cough and other signs of heavy respiratory infection, till death in some cases (Borovkova, cited in [25]). In pets, this parasite may cause bronchovesicular sounds, respiratory inflammation, sneezing, wheezing, chronic moist or dry cough, (broncho)-pneumonia respiratory failure and heavy parasite burdens may lead to mortality [10, 31]. More studies are also necessary to evaluate the actual role of *E. aerophilus* in causing undiagnosed respiratory distresses in companion dogs and cats.

Another topic which needs to be better elucidated is the actual role of *E. aerophilus* in causing lung diseases in humans. Indeed, only 12 cases of infection have been published in the literature [13], but it is likely that the infection is underdiagnosed because the clinical signs may overlap a plethora of respiratory diseases which may be self-limiting or may resolve after non-specific treatments.

## References

[1] Borgsteede FHM. 1984 Helminth parasites of wild foxes (*Vulpes vulpes* L.) in The Netherlands. Zeitschrift für Parasitenkunde, 70, 281–285 6741217 10.1007/BF00927813

[2] Bowman DD, Lynn RC, Eberhard ML. 2003 Georgi’s Parasitology for Veterinarians, 8th edn St. Louis: Sauders

[3] Butterworth EW, Beverley-Burton M. 1980 The taxonomy of *Capillaria* spp. (Nematoda: Trichuroidea) in carnivorous mammals from Ontario, Canada. Systematic Parasitology, 1, 211–236

[4] Chautan M, Pontier D, Artois M. 2000 Role of rabies in recent demographic changes in red fox populations in Europe. Mammalia, 64, 391–410

[5] Christenson RO. 1935 Studies on the morphology of the common fox lungworm, *Capillaria aerophila* (Creplin, 1839). Transactions of the American Microscopical Society, 54, 145–154

[6] Davidson RK, Gjerde B, Vikoren T, Lillehaug A, Handeland K. 2006 Prevalence of *Trichinella* larvae and extra-intestinal nematodes in Norwegian red foxes (*Vulpes vulpes*). Veterinary Parasitology, 136, 307–316 16378689 10.1016/j.vetpar.2005.11.015

[7] di Cesare A, Castagna G, Meloni S, Otranto D, Traversa D. 2012 Mixed trichuroid infestation in a dog from Italy. Parasites & Vectors, 5, 128 22731958 10.1186/1756-3305-5-128 PMC3414829

[8] di Cesare A, Castagna G, Otranto D, Meloni S, Milillo P, Latrofa MS, Paoletti B, Bartolini R, Traversa D. 2012 Molecular detection of *Capillaria aerophila*, an agent of canine and feline pulmonary capillariosis. Journal of Clinical Microbiology, 50, 1958–1963 22442326 10.1128/JCM.00103-12 PMC3372154

[9] di Cesare A, Otranto D, Latrofa MS, Meloni S, Castagna G, Morgan E, Lalosevic D, Mihalca A, Padre L, Traversa D. 2012 Genetic characterization of *Eucoleus aerophilus* from different hosts and countries. in: Proceedings of the 27th Conference of the Italian Society of Parasitology (SO.I.PA.), 26th–29th June, Alghero, Italy

[10] Foster S, Martin P. 2011 Lower respiratory tract infections in cats. Journal of Feline Medicine and Surgery, 13, 313–332 21515220 10.1016/j.jfms.2011.03.009 PMC7129729

[11] König A, Romig T, Thoma D, Kellermann K. 2005 Drastic increase in the prevalence of *Echinococcus multilocularis* in foxes (*Vulpes vulpes*) in southern Bavaria, Germany. European Journal of Wildlife Research, 51, 277–282

[12] Laloševic D, Dimitrijevic S, Jovanovic M, Klun I. 2001 Pulmonary aelurostrongylosis in cats. Veterinarski Glasnik, 55, 181–185 (in Serbian).

[13] Laloševic D, Laloševic V, Klem I, Stanojev-Jovanovic D, Pozio E. 2008 Pulmonary capillariasis miming bronchial carcinoma. American Journal of Tropical Medicine and Hygiene, 78, 14–16 18187778

[14] Magi M, Macchioni F, dell’Omodarme M, Prati MC, Calderini P, Gabrielli S, Iori A, Cancrini G. 2009 Endoparasites of red fox (*Vulpes vulpes*) in central Italy. Journal of Wildlife Disease, 45, 881–885 10.7589/0090-3558-45.3.881 19617506

[15] Miterpáková M, Hurnĺková Z, Antolová D, Dubinský P. 2009 Endoparasites of red fox (*Vulpes vulpes*) in the Slovak Republic with the emphasis on zoonotic species *Echinococcus multilocularis* and *Trichinella* spp. Helminthologia, 46, 73–79

[16] Moravec F. 1982 Proposal of a new systematic arrangement of nematodes of the family Capillariidae. Folia Parasitologica (Praha), 29, 119–132 7106653

[17] Moravec F. 2000 Review of capillariid and trichosomoidid nematodes from mammals in the Czech Republic and the Slovak Republic. Acta Societatis Zoologicae Bohemicae, 64, 271–304

[18] Moravec F, Prokopic J, Shlikas AV. 1987 The biology of nematodes of the family Capillariidae Neveu-Lemaire, 1936. Folia Parasitologica (Praha), 34, 39–56 3583129

[19] Nithikathkul C, Saichua P, Royal L, Cross JH. 2011 Capillariosis, in Oxford Textbook of Zoonoses, 2nd Edition, Biology, Clinical Practice, and Public Health Control, Palmer SR, Lord Soulsby EJL, Torgerson P, Brown DWG, Editors.Oxford University Press: Oxford p. 727–737

[20] Rajkovic-Janje R, Marinculic A, Bosnic S, Benic M, Vinkovic B, Mihaljevic Z. 2002 Prevalence and seasonal distribution of helminth parasites in red foxes (*Vulpes vulpes*) from the Zagreb County (Croatia). Zeitschrift für Jagdwissenschaft, 48, 151–160

[21] Reperant LA, Hegglin D, Fischer C, Kohler L, Weber JM, Deplazes P. 2007 Influence of urbanization on the epidemiology of intestinal helminths of the red fox (*Vulpes vulpes*) in Geneva, Switzerland. Parasitology Research, 101, 605–611 17393184 10.1007/s00436-007-0520-0

[22] Robardet E, Giraudoux P, Caillot C, Boue F, Cliquet F, Augot D, Barrat J. 2008 Infection of foxes by *Echinococcocus multilocularis* in urban and suburban areas of Nancy, France: influence of feeding habits and environment. Parasite, 15, 77–85 18416250 10.1051/parasite/2008151077

[23] Romashov BV. 2000 Three capillariid species (Nematoda, Capillariidae) of carnivores (Carnivora) and discussion of system and evolution of the nematode family Capillariidae. 1. Redescription of *Eucoleus aerophilus* and *E. boehmi*. Zoologichesky Zhurnal, 79, 1379–1391 (in Russian).

[24] Simpson VR. 2002 Wild animals as reservoirs of infectious diseases in the UK. The Veterinary Journal, 163, 128–146 12093188 10.1053/tvjl.2001.0662

[25] Skryabin KI, Shihobalova NP, Orlov IV. 1957 Trichocephalids and capillariids of animals and humans, and the disease caused by them, Fundamentals of nematodology, Vol VI Akademiya Nauk SSSR: Moscow p. 536–548 (in Russian).

[26] Soulsby EJL. 1982 Helminths, arthropods and protozoa of domesticated animals, 7th edition Philadelphia: Lea & Febiger

[27] Sréter T, Széll Z, Marucci G, Pozio E, Varga I. 2003 Extraintestinal nematode infections of red foxes (*Vulpes vulpes*) in Hungary. Veterinary Parasitology, 115, 329–334 12944046 10.1016/s0304-4017(03)00217-6

[28] Taylor MA, Coop RL, Wall RL. 2007 Veterinary parasitology, 3rd edn Oxford: Blackwell Publishing Ltd.

[29] Traversa D. 2012 Pet roundworms and hookworms:Acontinuing need for global warming. Parasites & Vectors, 5, 91 22574783 10.1186/1756-3305-5-91 PMC3418564

[30] Traversa D, di Cesare A, Milillo P, Iorio R, Otranto D. 2009 Infection by *Eucoleus aerophilus* in dogs and cats: is another extra-intestinal parasitic nematode of pets emerging in Italy? Research in Veterinary Science, 87, 270–272 19298989 10.1016/j.rvsc.2009.02.006

[31] Traversa D, di Cesare A, Conboy G. 2010 Canine and feline cardiopulmonary parasitic nematodes in Europe: emerging and underestimated. Parasites & Vectors, 3, 62 20653938 10.1186/1756-3305-3-62 PMC2923136

[32] Traversa D, di Cesare A, Lia RP, Castagna G, Meloni S, Heine J, Strube K, Milillo P, Otranto D, Meckes O, Schaper R. 2011 New insights into morphological and biological features of *Capillaria aerophila* (Trichocephalida, Trichuridae). Parasitology Research, 109 (suppl. 1), S97–S104 21739379 10.1007/s00436-011-2406-4

[33] Vos AC. 1995 Population dynamics of the red fox (*Vulpes vulpes*) after the disappearance of rabies in county Garmisch- Partenkirchen, Germany, 1987–1992. Annales Zoologici Fennici, 32, 93–97

